# Assessment of nerve involvement in the lumbar spine: agreement between magnetic resonance imaging, physical examination and pain drawing findings

**DOI:** 10.1186/1471-2474-11-202

**Published:** 2010-09-10

**Authors:** Bo C Bertilson, Eva Brosjö, Hans Billing, Lars-Erik Strender

**Affiliations:** 1 Center for Family and Community Medicine, Karolinska Institutet, Alfred Nobels allé 12, 141 83 Huddinge, Stockholm, Sweden; 2 Radiological department, Ersta Hospital, Fjällgatan 44 Södermalm, Box 4622, 116 91 Stockholm, Sweden

## Abstract

**Background:**

Detection of nerve involvement originating in the spine is a primary concern in the assessment of spine symptoms. Magnetic resonance imaging (MRI) has become the diagnostic method of choice for this detection. However, the agreement between MRI and other diagnostic methods for detecting nerve involvement has not been fully evaluated. The aim of this diagnostic study was to evaluate the agreement between nerve involvement visible in MRI and findings of nerve involvement detected in a structured physical examination and a simplified pain drawing.

**Methods:**

Sixty-one consecutive patients referred for MRI of the lumbar spine were - without knowledge of MRI findings - assessed for nerve involvement with a simplified pain drawing and a structured physical examination. Agreement between findings was calculated as overall agreement, the p value for McNemar's exact test, specificity, sensitivity, and positive and negative predictive values.

**Results:**

MRI-visible nerve involvement was significantly less common than, and showed weak agreement with, physical examination and pain drawing findings of nerve involvement in corresponding body segments. In spine segment L4-5, where most findings of nerve involvement were detected, the mean sensitivity of MRI-visible nerve involvement to a positive neurological test in the physical examination ranged from 16-37%. The mean specificity of MRI-visible nerve involvement in the same segment ranged from 61-77%. Positive and negative predictive values of MRI-visible nerve involvement in segment L4-5 ranged from 22-78% and 28-56% respectively.

**Conclusion:**

In patients with long-standing nerve root symptoms referred for lumbar MRI, MRI-visible nerve involvement significantly underestimates the presence of nerve involvement detected by a physical examination and a pain drawing. A structured physical examination and a simplified pain drawing may reveal that many patients with "MRI-invisible" lumbar symptoms need treatment aimed at nerve involvement. Factors other than present MRI-visible nerve involvement may be responsible for findings of nerve involvement in the physical examination and the pain drawing.

## Background

Detection of nerve involvement is considered a primary diagnostic concern in the assessment of spine disorders. This is because nerve involvement originating in the spine may indicate a need for invasive treatment and also because the resulting pain is often resistant to drug therapy and has less favourable recovery rates [1-5]. Magnetic resonance imaging (MRI) has emerged as the diagnostic method of choice for assessing spine disorders and especially for detecting nerve involvement [6-8]. MRI can be deemed to be too sensitive, with the risk of showing findings not associated with objective nerve involvement, which can lead to potentially harmful clinical measures including surgery [6]. However, scientifically, the agreement between MRI-visible nerve involvement and other diagnostic methods remains speculative, except that severe MRI-visible nerve involvement in the lumbar spine is associated with distal leg pain, which is considered a sign of nerve involvement [8-13]. Our experience is that MRI is rather insensitive in detecting nerve involvement found in a clinical examination. This study was motivated by the discrepancy between the notion that MRI is sensitive, the science, and our experience of MRI in the assessment of lumbar spine disorders. We recognize that treatment, patient reliability and insurance questions ultimately depend largely on radiological assessment, especially the detection of MRI-visible nerve involvement [8].

The aim of this diagnostic study on 61 patients with long-standing nerve root symptoms referred for MRI of the lumbar spine was to evaluate the agreement between MRI-visible nerve involvement and findings of nerve involvement detected in a structured physical examination and in a simplified pain drawing. We present novel data on the prevalence, sensitivity, specificity and positive and negative predictive value of MRI-visible nerve involvement in relation to findings of nerve involvement detected in the physical examination and the pain drawing.

## Methods

### Setting and participants

From February to September 2004, all patients 18-80 years of age referred to Ersta radiological clinic in Stockholm for MRI of some part of the spine were invited to participate. A written invitation with information about the study was extended to 123 consecutive patients. Exclusion criteria were previous back surgery, life-threatening disease, inability to speak Swedish or patient known by the conductor of the physical examination. Twenty patients were thereby excluded from the study. Three more were excluded during the study; one because of lack of time, one because of claustrophobia, and one who was outside the age limit. Among the remaining 100 patients, 61 were examined with MRI of the lumbar spine and the other 39 with MRI of the cervical or thoracic spine. Only data from the 61 patients with MRI of the lumbar spine are evaluated in this paper. Data from the other patients are evaluated in a separate paper.

Referring physicians were general practitioners, orthopaedic surgeons and pain specialists working in outpatient care, who were informed about the study before their patients were invited to participate. The pain drawing, the history and the physical examination were assessed by one of the authors who has specialised in orthopaedic medicine for 20 years. Two certified radiologists each with about 10 years' experience of spinal MRI performed assessment of the MRI. Informed consent was obtained to present patient data anonymously. The southern ethical board of the Karolinska Institutet approved the study on the 8^th ^of December 2003.

### Procedure, protocols and technique

#### A first assessment based on a simplified pain drawing

The pain drawing (Additional file 1) was mailed to each patient a few days before the MRI scan with instructions to fill it out at home and bring it to the MRI clinic 45 minutes prior to the appointment. On arrival to the clinic, the patient handed the pain drawing to the examiner, who made a written statement of his initial impression assessment of the drawing before any other information from the referring physicians or the patient was made known to him. A neuroanatomical distribution pattern of discomfort indicating nerve involvement [14,15] originating in the spine was assessed subjectively to answer the following questions about nerve involvement: yes/no, right/left/bilateral, grade 1 or 2 and suspected spine segment(s). Grade 2 indicated more severe nerve involvement than grade1 and was subjectively assessed based on the darkness of the shading on the pain drawing. History questionnaires were then filled out.

#### A second assessment based on a structured physical examination

The physical examination was performed during the half hour preceding the MRI scan. It focused on the detection of nerve involvement originating in the spine. A protocol (Additional file 2) prepared by the examiner and based on his standard examination method was filled out for each patient and assessed as described in the protocol. The dermatome map by Netter [15] was used to guide the assessment of sensibility to touch and pain in different areas of the body. All sensibility tests were done bimanually at right and left side simultaneously from the chin (C2 nerve) to the lateral part of the foot (S1 nerve)

#### A third assessment based on MRI

MRI was performed using a 1.0 Tesla scanner (Philips Intera) with a dedicated phased array spinal coil to produce sagittal and axial T1 and T2 spin and turbo spin echo sequences (slice thickness 3 mm, interslice gap 0.3 mm, fields of view 25 cm for sagittal and 16 cm for axial images). A protocol (Additional file 3) prepared by the radiologists and based on their standard examination method was filled out for each patient. Findings were assessed by two independent radiologists and graded as noted in the protocol or as described below in additional definitions that were determined before the start of the study. Visible nerve involvement resulting from spine processes was assessed subjectively. Each radiologist made a first assessment before reading the patient's history from the referring physician. Each radiologist also made a second assessment after reading the patient's history from the referring physician. The immediately preceding assessment of the pain drawing and the physical examination were not made known to the radiologists at any time.

#### Additional definitions not noted in the MRI protocol

Disc water content - decreased; *slight *= grey or light black disc and *significant *= very dark or black. Disc height - decreased; *slight *= <50% decreased height and *significant *= >50%. HIZ (high intensity zone); *present *defined as bright disc rim sign indicative of annular tear. Spinal stenosis; *slight *= transverse dural area >0.7< 1.2 cm^2 ^and *significant *< 0.7 cm^2^. Protrusion/protuberance grade extraforaminal; *slight *= reaching to nerve and *significant *= deranging nerve. Protrusion/protuberance grade foraminal; *slight *= <50% decreased width and *significant *= >50%. Restriction type by disc; *bulging *= broad-based bulging of the disc beyond the vertebral disc margin with intact annulus fibrosus (usually < 3 mm) and *hernia *= focal protrusion of the disc through a defect in the annulus fibrosus into the spinal canal, foraminal or lateral space (usually > 3 mm). Findings of significant spinal stenosis were considered as findings of nerve involvement.

### Analytical methods

Graded findings were dichotomised in two steps: (1) all positive findings irrespective of grade were recorded as yes and the rest as no, and (2) all positive findings of grade 2 or more were recorded as yes and the rest as no.

Inter-examiner reliability in the detailed findings of the MRI assessment was evaluated using kappa statistics and classified as suggested by Altman: <0 worse than chance, 0-0.2 poor, 0.21-0.4 fair, 0.41-0.6 moderate, 0.61-0.8 good and >0.8 very good [16].

A one-sample test of proportions was used to compare prevalence of findings. Agreement between MRI-visible nerve involvement and findings of nerve involvement in the pain drawing and the physical examination was calculated as overall agreement and with McNemar's exact significance probability test for bias in matched pair data [16]. A p value of <0.05 was considered statistically significant. Sensitivity, specificity, and positive and negative predictive values of MRI-visible nerve involvement to findings of nerve involvement in the pain drawing and the physical examination were also calculated.

STATA for Windows version 9.2 was used for calculations.

## Results

### Patient characteristics (Table 1)

**Table 1:** Patient characteristics (n = 61)

	Median or %
Basic characteristics	
Age, years (range)	60 (27-80)
Gender, % females	49
Length, cm (range)	173 (155-195)
Weight, kg (range)	80 (52-115)
Body mass index (range)	26 (20-40)
Born in Sweden, %	75
Current smoker, %	23
Believes MRI diagnose*, %	100
Desires operation†, %	85
Discomfort in low back region	
Debut, years ago (range)	14 (0-50)
Presently, %	89
Duration <3 months, %	21
Duration 3-12 months, %	23
Duration 1-2 years, %	8
Duration > 2 years, %	48
Discomfort into leg‡/foot region	
Presently, %	56
Duration <3 months, %	23
Duration 3-12 months, %	25
Duration 1-2 years, %	8
Duration > 2 years, %	44

* MRI will present explanation to their discomfort

† If MRI show surgical treatable explanation

‡ Below the buttock

Median age was 60 years and 49% were women. Every patient (100%) believed that MRI examination would provide an explanation of their discomfort and 85% desired an operation if there was a surgically treatable explanation. Median time since debut of low back pain was 14 years and 44% experienced discomfort into the leg/foot region since more than 2 years.

### Inter-examiner reliability

Inter-examiner reliability in the assessment of nerve involvement was generally moderate to good in segments L2-3 and L3-4 and poor to fair in segments L4-5 and L5-S1. The radiologists differed, though not significantly, in their assessments; the one who noted more MRI-visible nerve involvement is the one whose data are used for comparison in this paper. When calculations were made from the assessments by the radiologist who noted less visible nerve involvement, the overall results given below were not altered. These assessments were not changed after the radiologists read the patient histories.

### Prevalence of findings (Table 2)

**Table 2:** Prevalence of findings in the MRI, the physical examination and pain drawing in assessing the lumbar spine (n = 61)

		Magnetic resonance image																Physical examination								Pain drawing	
		**Visible nerve involvement**		**Decreased**				**High intensity zone**		**Increased medulla signal**		**Spinal stenosis**		**Disco-ligament protrution**		**Bone pro- tuberance**		**Disturbed**								**Indicating nerve involvement**	
																								
				**disc water**		**disc height**												**sensibility**				**reflex**		**motor**			
																								
																		**to touch**		**to pain**		**function**		**function**			
				
				**Grade**		**Grade**		**Grade**		**Grade**		**Grade**		**Grade**		**Grade**		**Grade**		**Grade**		**Grade**		**Grade**		**Grade**	
		
**Segment (nerve)**	**Location**	**Patients**		**1**	**2**	**1**	**2**	**1**	**2**	**1**	**2**	**1**	**2**	**1**	**2**	**1**	**2**	**1**	**2**	**1**	**2**	**1**	**2**	**1**	**2**	**1**	**2**
		**no**	**%**	**%**	**%**	**%**	**%**	**%**	**%**	**%**	**%**	**%**	**%**	**%**	**%**	**%**	**%**	**%**	**%**	**%**	**%**	**%**	**%**	**%**	**%**	**%**	**%**

T 11-12 (T12)	right	0	0															7	2	10	3	2	0			0	0
	left	0	0															5	2	13	7	2	0			0	0
	bilateral	0	0															2	2	5	2	2	0			0	0
	any	0	0	11	2	3	0	0	0	0	0	0	0	0	0	0	0	10	2	18	8	2	0			0	0
T 12-L1 (L1)	right	0	0															10	3	26	5					0	0
	left	0	0															15	2	36	8					0	0
	bilateral	0	0															3	2	11	2					0	0
	any	0	0	8	3	2	0	0	0	0	0	0	0	2	2	0	0	21	3	51	11					0	0
L 1-2 (L2)	right	0	0															15	2	20	3			28	3	0	0
	left	0	0															16	2	31	10			31	7	3	0
	bilateral	0	0															3	0	8	0			13	0	0	0
	any	0	0	20	10	16	3	2	0	0	0	2	0	5	0	0	0	28	3	43	13			46	10	3	0
L 2-3 (L3)	right	1	2															13	0	20	3			18	2	2	0
	left	2	3															21	2	31	8			20	5	0	0
	bilateral	1	2															5	0	10	2			10	0	0	0
	any	2	3	33	10	23	5	2	0	0	0	8	0	13	0	8	7	30	2	41	10			28	7	2	0
L 3-4 (L4)	right	7	11															10	0	18	3	31	23	31	3	11	0
	left	5	8															18	2	38	8	33	21	31	7	8	0
	bilateral	4	7															5	0	10	2	28	20	15	0	7	0
	any	8	13	41	10	28	3	5	0	0	0	18	7	30	3	8	8	23	2	46	10	36	25	48	10	13	0
L 4-5 (L5)	right	11	18															20	7	25	10	46	25	34	15	52	10
	left	13	21															26	5	41	13	41	25	49	21	59	3
	bilateral	6	10															5	2	10	2	38	25	26	11	38	0
	any	18	30	79	21	49	10	10	0	0	0	23	8	49	8	13	10	41	10	56	21	49	25	57	25	74	13
L 5-S1 (S1)	right	4	7															15	5	18	7	36	23	16	2	21	3
	left	7	11															21	3	30	7	46	23	34	10	23	3
	bilateral	0	0															3	0	7	0	34	21	13	2	15	2
	any	11	18	72	39	56	33	2	0	0	0	2	0	48	18	13	10	33	8	41	13	48	25	38	10	30	5
T11-S1	any	30	49	93	52	85	44	15	0	0	0	30	11	79	28	30	21	57	18	77	39	66	31	80	38	95	16

Grade 1 includes all positive findings, grade 2 includes all positive findings grade 2 or more; T = thoracic; L = lumbar; S = sacral

MRI-visible nerve involvement at any location and segment was significantly less prevalent than all grade 1 physical examination and pain drawing findings of nerve involvement except for sensibility to touch (p = 0.207). MRI, physical examination and pain drawing findings of grade 2 (or more) were significantly less prevalent than findings of grade 1 except for medulla cord signal and bone protuberance.

#### MRI findings

Nerve involvement was found in 30 patients (49%) in a total of 50 locations, most prevalent in segment L4-5 and in more than one segment in 8 patients (27%). In segments T11 to L3 there were a total of 3 MRI-visible nerve involvements making calculations on agreement, sensitivity and specificity etcetera less meaningful. However, the data and calculations on these segments are presented in additional file 4 and 5.

The most prevalent disc pathology finding was decreased water content and the most prevalent space-restricting finding was discoligament protrusion; 93% and 79%, respectively, had these findings of at least grade 1 in any one segment. The least prevalent finding was medulla cord signal with 0%. All MRI findings were most prevalent in segments L4-S1 and least prevalent in segments T11-L1. Decreased disc water content and height as well as discoligament protrusion occurred in more than one segment in more than 50% of the patients (data not shown).

#### Physical examination findings of nerve involvement

Disturbed motor function was the most prevalent finding and disturbed sensibility to touch the least; 80% and 57%, respectively, had these findings of at least grade 1 in any one segment. All physical examination findings were most prevalent in segment L4-5 and least in segment T11-12. Physical examination findings of every kind were present in more than one adjacent segment in more than 50% of the patients (data not shown).

#### Pain drawing findings of nerve involvement

A neuroanatomical distribution pattern of discomfort indicating nerve involvement originating in the spine was found in 95% of the patients. These findings were most prevalent for segment L4-5 and least for segments T11-L1. They included more than one adjacent segment in 24% of the patients (data not shown).

### Agreement between MRI-visible nerve involvement and findings of nerve involvement detected in the physical examination and pain drawing (Table 3)

**Table 3:** Agreement between MRI-visible nerve involvement and findings of nerve involvement detected in the physical examination and pain drawing in assessing the lumbar spine (n = 61)

		MRI		Physical examination								Pain drawing	
	
		Visible nerve involvement		Disturbed sensibility				Disturbed reflex function		Disturbed motor function		Indicating nerve involvement	
										
				to touch		to pain							
		
				Agreement		Agreement		Agreement		Agreement		Agreement	
Segment (nerve)	Location	Patients		Overall	McN	Overall	McN	Overall	McN	Overall	McN	Overall	McN
		no	%	%	P	%	P	%	P	%	P	%	P
	Dichotomised findings irrespective of grade in the physical examination and pain drawing												
L 3-4 (L4)	right	7	11	79	°	70	°	64	0.017	67	0.012	77	°
	left	5	8	74	°	54	0.001	66	0.002	64	0.004	84	°
	bilateral	4	7	89	°	84	°	69	0.004	79	°	87	°
	any	8	13	67	°	41	0.001	61	0.007	52	0	74	°
L 4-5 (L5)	right	11	18	69	°	67	°	62	0.001	61	°	49	0
	left	13	21	62	°	54	0.036	54	0.036	56	0.002	39	0
	bilateral	6	10	89	°	84	°	59	0.001	70	0.031	56	0.002
	any	18	30	43	°	41	0.011	57	0.029	46	0.005	43	0
L 5-S1 (S1)	right	4	7	82	°	79	°	64	0	80	°	79	0.023
	left	7	11	74	°	66	0.027	46	0	64	0.004	75	°
	bilateral	0	0	97	°	93	°	66	0	87	0.008	85	0.004
	any	11	18	62	°	54	0.013	44	0.003	57	0.029	72	°
T11-S1 mean of	any			69		54		65		56		83	

	Dichotomised findings of grade 2 or more in the physical examination and pain drawing												
L 3-4 (L4)	right	7	11	89	0.016	85	°	72	°	85	°	89	0.016
	left	5	8	90	°	84	°	74	°	85	°	92	°
	bilateral	4	7	93	°	92	°	77	°	93	°	93	°
	any	8	13	85	0.039	77	°	69	°	77	°	87	0.008
L 4-5 (L5)	right	11	18	75	°	75	°	64	°	70	°	75	°
	left	13	21	74	0.021	69	°	64	°	70	°	75	0.007
	bilateral	6	10	89	°	89	°	69	°	82	°	90	0.031
	any	18	30	61	0.023	56	°	59	°	59	°	64	°
L 5-S1 (S1)	right	4	7	92	°	90	°	74	0.021	92	°	90	°
	left	7	11	85	°	85	°	66	°	85	°	89	°
	bilateral	0	0	100	°	100	°	79	0	98	°	98	°
	any	11	18	77	°	75	°	61	°	79	°	80	0.039
T11-S1 mean of	any			87		80		72		79		90	

McN = p-value for exact Mc Nemar significance probability test; T = thoracic; L = lumbar; S = sacral; ° = non significant p-value (> 0.05)

#### Considering findings irrespective of grade in the physical examination and pain drawing

The mean overall agreement for MRI-defined nerve involvement at any location in segments T11-S1 ranged from 54 to 83%, with a pain drawing indicating nerve involvement showing the best agreement with MRI-visible nerve involvement. The lowest overall agreement for all tests (41-57%) was observed in segment L4-5. McNemar's test showed significant bias between MRI and physical examination findings of nerve involvement in most segments, except disturbed sensibility to touch where there was no bias in the three lower segments. McNemar's test showed significant differences between MRI and pain drawing findings of nerve involvement in segments L4-5 and L5-S1.

#### Considering findings of grade 2 or more in the physical examination and pain drawing

The mean overall agreement for MRI-defined nerve involvement at any location in segments T11-S1 ranged from 72 to 90%, with a pain drawing indicating nerve involvement showing the best agreement with MRI-visible nerve involvement. For disturbed sensibility to touch, pain and disturbed motor function, the overall agreement for findings of grade 2 or more was significantly greater (respectively p = 0.002, p = 0.000 and p = 0.000) than the overall agreement for findings irrespective of grade. The lowest overall agreement for all tests (56-64%) was observed in segment L4-5. McNemar's test showed a significant difference between MRI and physical examination/pain drawing findings of nerve involvement in less than 20% of all locations.

### Sensitivity, specificity, and positive and negative predictive values of MRI-visible nerve involvement to findings of nerve involvement detected in the physical examination and pain drawing (Table 4)

**Table 4:** Sensitivity, specificity, and positive and negative predictive values of MRI-visible nerve involvement to findings of nerve involvement detected in the physical examination and pain drawing in assessing the lumbar spine (n = 61)

		MRI		Physical examination																Pain drawing			
		**Visible nerve involvement**		**Disturbed sensibility**								**Disturbed reflex function**				**Disturbed motor function**				**Indicating nerve involvement**			
																
				**to touch**				**to pain**															
		
**Segment (nerve)**	**Location**	**Patients**		**Se**	**Sp**	**PPV**	**NPV**	**Se**	**Sp**	**PPV**	**NPV**	**Se**	**Sp**	**PPV**	**NPV**	**Se**	**Sp**	**PPV**	**NPV**	**Se**	**Sp**	**PPV**	**NPV**
		**no**	**%**	**%**	**%**	**%**	**%**	**%**	**%**	**%**	**%**	**%**	**%**	**%**	**%**	**%**	**%**	**%**	**%**	**%**	**%**	**%**	**%**

				Dichotomised findings irrespective of grade in the physical examination and pain drawing																			
																							
L 3-4 (L4)	right	7	11	0	87	0	89	0	86	0	80	11	88	29	69	16	90	43	70	0	87	0	87
	left	5	8	0	90	0	80	0	87	0	59	10	93	40	68	5	90	20	68	0	91	0	91
	bilateral	4	7	0	93	0	95	0	93	0	89	6	93	25	72	0	92	0	84	0	93	0	93
	any	8	13	7	85	13	75	0	76	0	47	14	87	38	64	14	88	50	53	0	85	0	85
L 4-5 (L5)	right	11	18	17	82	18	80	20	83	27	76	29	91	73	60	19	83	36	66	19	83	55	48
	left	13	21	19	78	23	73	20	78	38	58	20	78	38	58	27	84	62	54	17	72	46	38
	bilateral	6	10	33	91	17	96	17	91	17	91	9	89	33	62	13	91	33	75	4	87	17	60
	any	18	30	16	61	22	51	24	63	44	40	37	77	61	56	29	69	56	42	31	75	78	28
L 5-S1 (S1)	right	4	7	11	94	25	86	9	94	25	82	9	95	50	65	10	94	25	84	15	96	50	81
	left	7	11	15	90	29	80	11	88	29	70	4	82	14	50	14	90	43	67	21	91	43	80
	bilateral	0	0	†	100	†	97	†	100	†	93	†	100	†	66	†	100	†	87	†	100	†	85
	any	11	18	20	83	36	68	16	81	36	58	10	75	27	48	17	82	36	62	33	88	55	76
T11-S1 mean of	any			11	89	18	72	10	88	20	56	20	85	42	67	15	87	36	56	16	92	33	83

Se = sensitivity; Sp = specificity; PPV/NPV = positive/negative predictive value; T = thoracic; L = lumbar; S = sacral; † = no finding in the physical examination/pain drawing

#### Considering findings irrespective of grade in the physical examination and pain drawing

The mean sensitivity for MRI-visible nerve involvement to the presence of physical examination and/or pain drawing findings of nerve involvement at any location in segments T11-S1 ranged from 10 to 20% depending on the test; sensitivity was lowest for disturbed sensibility to pain or touch and highest for disturbed reflex function. This means that at best 1 out of 5 and at worst 1 out of 10 patients with a physical examination finding indicating nerve involvement originating in the lumbar spine were considered as MRI-visible nerve involvement. Sensitivity was lower in the upper segments and higher in segment L4-5 (at any location), where 16-37% of the positive physical examination/pain drawing findings were considered as MRI-visible nerve involvement.

The mean specificity for MRI-visible nerve involvement to the absence of physical examination and/or pain drawing findings of nerve involvement at any location in segments T11-S1 ranged from 85-92%. Specificity was lowest for the absence of disturbed reflex function and highest for the absence of a pain drawing indicating nerve involvement. Specificity was lower in segment L4-5 (at any location 61-77%) and higher in the upper segments. This means that about 1/3 to 1/4 of those having no physical examination or pain drawing finding indicating nerve involvement in segment L4-5 were considered as having MRI-visible nerve involvement in segment L4-5.

The mean positive predictive value for MRI-visible nerve involvement at any location in segments T11-S1 ranged from 18 to 42%. The lowest value was for disturbed sensibility to touch and the highest for disturbed reflex function. The positive predictive value in segment L4-5 (at any location) ranged from 22 to 78%, with the lowest value for disturbed sensibility to touch and the highest for a pain drawing indicating nerve involvement.

The mean negative predictive value for MRI-visible nerve involvement at any location in segments T11-S1 ranged from 56 to 83%. The lowest value was no disturbed sensibility to pain and no disturbed motor function, and the highest for a pain drawing indicating no nerve involvement. The negative predictive value in segment L4-5 (at any location) ranged from 28 to 56%; the lowest value for a pain drawing indicating no nerve involvement and the highest for no disturbed reflex function.

#### Considering findings of grade 2 or more in the physical examination and pain drawing (data not shown)

With one exception, the mean sensitivity and specificity for MRI-visible nerve involvement at any location in segments T11-S1 did not differ significantly from the observations made irrespective of grade in the physical examination and pain drawing. The mean positive and negative predictive values for MRI-visible nerve involvement at any location in segments T11-S1 respectively decreased and increased significantly for all tests of grade 2. This means that MRI-visible nerve involvement was significantly less often confirmed as a grade 2 than a grade 1 positive physical examination/pain drawing finding. Also, patients with no MRI-visible nerve involvement lacked a grade 2 positive physical examination/pain drawing finding significantly more often than a grade 1.

## Discussion

The results of this diagnostic study on patients with long-standing nerve root symptoms referred for MRI of the lumbar spine showed that MRI-visible nerve involvement was significantly less prevalent than, and showed weak agreement with, physical examination and pain drawing findings of nerve involvement. The sensitivity, specificity, and positive and negative predictive values of MRI-visible nerve involvement to findings of nerve involvement detected in the structured physical examination and/or the simplified pain drawing do not support the notion that MRI is the diagnostic method of choice for detecting nerve involvement originating in the spine.

The mean sensitivity of MRI-visible nerve involvement to a positive neurological test of any degree was at best 20% for disturbed reflex function and at worst 10% for disturbed sensibility to pain. This means that for most patients, nerve involvement detected in a physical examination and/or pain drawing may go undetected if the clinician believes the MRI assessment of nerve involvement. This observation contrasts with the views of experts who suggest that the (greater) sensitivity of MRI in detecting spinal disorders can lead to potentially harmful clinical measures, for example surgery [6]. Roland et al. even suggest that a radiological report should include a caution to the effect that the clinician, and in the end the patient, should not worry about abnormalities seen on the MRI [17]. Such suggestions may explain why patients with back pain generally have very high expectations of the diagnostic value of MRI, at least believing that it will not fail to find the explanation for their discomfort, and therefore desire to have a scan. In our patient sample, 100% believed the MRI would (finally) provide the explanation for their discomfort, which for the median patient had started 14 years earlier and had plagued them to the extent that 85% desired back surgery after having been through years of conservative treatment. About half of these patients - and the referring clinicians - in the end received radiological reports that showed no visible nerve involvement. In accordance with clinical practice, this usually means that the patient's discomfort is labelled "non-specific" and therefore continues to receive conservative treatment and/or is referred for psychosocial treatment strategies.

Our study adds to earlier studies that have shown a weak agreement between MRI-visible nerve involvement and other findings of nerve involvement in the assessment of the lumbar spine [8,9,13,18,19]. One possible explanation to the weak agreement is that the standard MRI recording is made with the patient in supine position with minimal axial loading of the lumbar spine and consequently reasonably less bulging of discs. Another possible explanation to the weak agreement is that previous disc herniation caused pressure damage and pathologies other than standard MRI-visible nerve involvement can elicit nerve involvement. Studies have shown that inflammatory cytokines from leaking discs, functional instability and fluctuating disc bulges and restrictions caused by discoligament injuries, some visible on functional MRI, can elicit radiating pain and nerve involvement from the spine [20-25]. One hypothesis is that even minor MRI findings should be considered potential causes of nerve involvement. Our results give some support to this hypothesis as we observe that the prevalence of MRI findings of decreased disc water and height and protrusion, and of the physical examination findings of disturbed sensibility to pain and disturbed motor function and pain drawing findings of nerve involvement were in the same quartile (between 77 and 95%).

The correlation between MRI visible findings and treatment outcome has been studied. A contained herniated/bulging disc (not necessarily with visible nerve involvement) has been found indicative of a negative outcome in conservative treatment of lumbar radiculopathy [26,27]. On the contrary, a broad-based, extruded or sequestered disc is not indicative of a negative outcome [26,27]. This may be due to the well-known fact that the latter types of disc herniations are prone to be resorbed as opposed to the contained bulging disc [28-30]. We agree with Jensen et al saying; 'The time has come for clinicians to take the consequences of the fact that the "size" does matter, meaning that the more "prominent" the herniation is, the better..." and that "Pressure on the nerve root is not the crux of the matter" [27,30] even though some patients with acute cauda equina need prompt surgical intervention.

The correlation between clinical or other findings and treatment outcome has also been studied. Komori et al found that sensory disturbance was the most significant predicting factor of a negative conservative treatment outcome of lumbar radiculopathy [26]. On the other hand, the often used straight leg raising nerve tension test showed no significant correlation with treatment outcome [26]. Correlation between clinical findings of nerve involvement and a negative outcome of surgery of lumbar disc hernia has also been shown, indicating the need for careful pre- and postoperative neurological examination [31-33]. Laboratory findings like higher levels of neurofilament protein in the cerebrospinal fluid before surgery does also correlate to post-operative sequelae [34]. Neurofilament protein in the cerebrospinal fluid may indicate permanent damage of axons and Schwann cells in the affected nerve root and this may explain why about 1/3 of patients have persisting sensory and/or reflex deficits one year post-operatively while motor function tends to improve to a greater extent [32,35,36]. Weber found that 35% of patients with lumbar disc herniation still had sensory dysfunctions demonstrable 10 years after treatment. These observations are in line with our findings of sensory, reflex and motor function grade 2 deficits in about 1/3 of our patients and add emphasise to the need for more careful neurological examination and development of new treatment strategies for neuropathic pain which often does not resolve with time [5].

We do agree with the notion that an MRI report, made and/or read by less updated personnel, can lead to potentially harmful clinical measures, but the reason is not a high but rather a low sensitivity of MRI in detecting nerve involvement. It may be that the need for specific medication against nerve involvement and even invasive methods would be indicated more often if the low sensitivity of MRI in detecting nerve involvement observed in this study was considered [37]. Other potentially harmful clinical consequences if there is no MRI-visible nerve involvement include the diagnosis "non-specific" given to 80-90% of all patients with lumbar spine disorders, which may be translated by some colleagues and insurance personnel into dysfunctional, somatic, psychosomatic or even "non-existent". Such a diagnosis is often considered an insult by the patient suffering spinal pain and may leave him or her without hope for the future [38]. Non-specific pain *per se *may be more detrimental than pain due to a known cause and may contribute to the poor outcome of low back pain treatment [39,40]. Better diagnostic and treatment strategies and antidotes are needed for patients who may suffer physically and mentally from today's "MRI-invisible" symptoms [41].

An interesting observation is that findings of disturbed sensibility to touch and pain often occurred in more than one single dermatome. Chen has reported similar findings in patients with cervical disc protrusions [42]. In our study, this observation also applied to motor and reflex findings. A possible explanation is that a discoligament injury causing biochemical and/or mechanical stress to a nerve can initiate motor-sensory axonal neuropathy, which can progress both distally and proximally along nerve tracts [43,44]. The observation of widespread sensory and motor dysfunction seems to be related to the idea of sensitization. However, our patient sample generally had findings of nerve involvement spread from one injured spinal segment to surrounding areas and not to the whole body. Further studies are needed to explore possible mechanisms [45-47].

**Figure 1:**
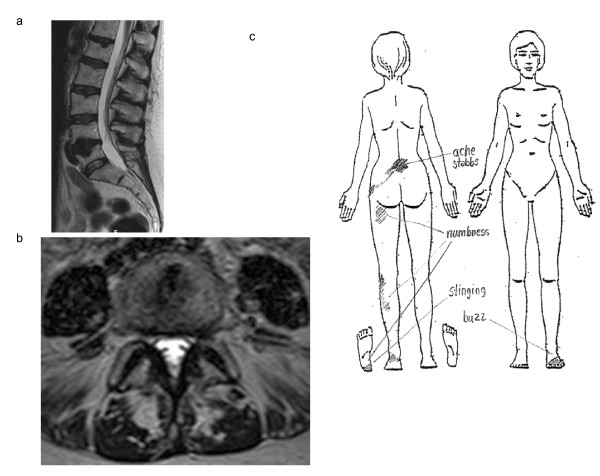
**No MRI-visible nerve involvement though obvious physical examination findings**. Figure 1ab: MRI of the lumbar spine of a woman aged 61 years. T2-weighted sagittal (a) and axial L4-5 (b) scans. The radiological assessment noted no visible nerve involvement but a slight paramedial disc protrusion at level L4-5, where an intraosseous disc hernia was also seen. Figure 1c: Pain drawing made by the patient in figure 1ab. The initial impression assessment of the pain drawing was that she had a left-sided L5 radiculopathy. Physical examination findings included: sensibility to touch and pain impaired in the lateral part of the left calf and slightly impaired in the whole left leg and lower left side of the trunk; tibialis posterior reflexes absent bilaterally and Achilles and patellar reflexes impaired bilaterally; motor function impaired for big toe extension and flexion on the left side. Patient history included 23 years of chronic backache, heel and Achilles pain (left side) and also urinary incontinence. Symptoms were initially acute when she fell from 3 meters and landed on her back. Standing, lifting, sitting and other axial loading of the spine increased her symptoms. Lying down relieved her symptoms.

**Figure 2:**
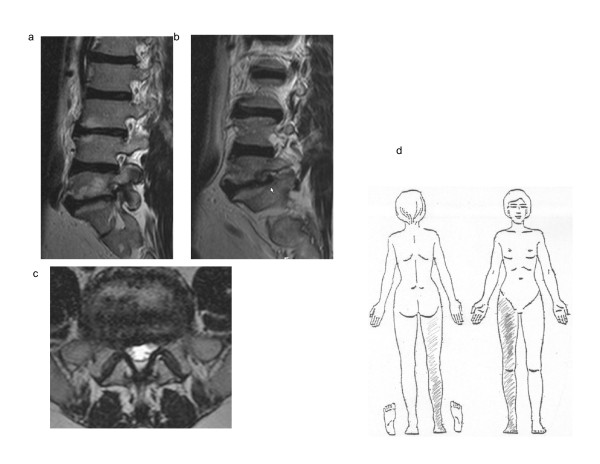
**MRI visible nerve involvement though no physical examination findings**. Figure 2abc: MRI of the lumbar spine of a man aged 48 years. T2-weighted sagittal right foraminal (a), sagittal right lateral (b) and axial L5-S1 (c) scans. The radiological assessment noted visible nerve involvement bilaterally at L5 and right-sided nerve involvement at S1 due to disc hernia and intervertebral arthrosis. Figure 2d: Pain drawing made by the patient in figure 2abc. The initial impression assessment of the pain drawing was that he had right-sided L4-S1 radiculopathy. No pathological findings were observed in the physical examination except that the right plantar reflex was slightly impaired. Patient history included 9 years of walking difficulties but no back pain. Symptoms started when he carried a heavy weight. Walking or standing still for 5 minutes made his right leg cramp and feel numb, like "lots of lactic acid". Bending forward relieved the pain.

Figure 1 exemplifies a case in which the radiologist noted no MRI-visible nerve involvement but the physical examination and pain drawing did, i.e. the specificity of MRI in determining nerve involvement was questionable. The physical examination findings also showed widespread disturbed sensibility in dermatomes adjacent to the most affected dermatome (L5). Figure 2 exemplifies a case in which MRI-visible nerve involvement was observed but there were almost no physical examination findings of nerve involvement, i.e. the specificity of MRI in determining nerve involvement was questionable.

### Limitations

The lack of a gold standard for detecting nerve involvement with which MRI and other diagnostic methods may be compared in the assessment of lumbar spine disorders is a major limitation [48]. Electro-diagnostic procedures are sometimes used as a gold standard for detecting nerve involvement. However, conventional electro-diagnostic procedures leave the function of small-calibre afferent fibres unexplored and therefore cannot identify the basis for positive sensory findings [49-52]. Even quantitative sensory testing, which is considered to include small fibre testing, seems to underestimate the prevalence of disturbed sensibility to touch in patients with partial nerve injury [53]. Myelography, discography and/or root blocks are used to confirm symptomatic discs and/or roots but these procedures have been less thoroughly evaluated and may not be considered gold standards for detecting nerve involvement [54]. In this study we chose the simple yet thorough physical examination and pain drawing methods for detecting nerve involvement originating in the spine and for comparison with MRI findings. This method for detecting nerve involvement has been suggested by The International Association for the Study of Pain [50,55].

The reliability and validity of these diagnostic methods must therefore be considered. For that purpose we performed reliability studies on clinical tests prior to this study. In those prior studies we found the highest inter-examiner reliability (kappa 0.67 with known patient history) for the bimanual sensibility to pain test with spurs described by Bertilson et al. [56,57]. Furthermore, validity was indirectly tested in another study where sensibility findings were compared to pain drawing patterns of nerve involvement and a 90% concordance with affected nerves was found [58]. Other studies on clinical tests in the assessment of nerve involvement have come to similar results; sensory testing generally shows good reliability as do motor function tests, while reflex function seems less reliable [59,60]. Inter-examiner reliability in various assessment of the pain drawing has been studied and generally been shown to be good [61-63]. The validity of the pain drawing has also been tested and 70-90% concordance with myelographic, computer tomographic/discographic and per-operative findings of disc pathology in the lumbar spine have been found [64-68]. Rankine when comparing pain drawing assessment of nerve root compression with MRI visible nerve root compression found that the pain drawing correctly classified only 58% of the patients [69]. However, the method used by Rankine to assess the pain drawing for nerve involvement by dividing up the lower body in regions was not the same as we used (assessing neuroanatomical distribution patterns like dermatome, myotome and/or sclerotome distribution of symptoms). Our results may therefore not be comparable.

Another limitation to consider is varying inter-examiner reliability in MRI assessment. However, similar reliability has been reported previously [70,71]. We present calculations based on the data from the radiologist who noted more (but not significantly more) visible nerve involvement. We refrain from presenting the other radiologist's assessments, as they would clutter the paper with data without adding any new insight.

Further limitations include the fact that the amount of statistical analyses makes possible that mass significance can influence the results. However, the difference in prevalence between findings of nerve involvement in the MRI and the pain drawing and the physical examination are not influenced by mass significance and these are the main findings upon which all other analyses are based.

Also a limitation is that the same examiner made the assessment of the pain drawing and the physical examination - having the pain drawing in memory - which could theoretically have influenced the physical examination findings. However, a prior study showed that knowledge of history and the pain drawing did not influence physical examination findings significantly [56].

A limitation to the generalisation of our results is that our patient sample had long-standing symptoms of low back pain (median debut 14 years prior) and radiation into the leg (44% > 2 years). However, patients with long-standing nerve root symptoms are those who rely most on MRI results for their future treatment; understanding and welfare, so we consider them most appropriate to study.

We acknowledge that normally a diagnosis is made on the basis of a sum of observations with varying reliability and validity. However, in this study we wanted to specifically compare a few of the main diagnostic methods as we consider that these methods - especially the MRI - are more commonly relied upon in the overall diagnostic conclusion.

### Future studies

Future studies may evaluate the agreement of other MRI findings of different grades and preferably more functional MRI recordings (for example while standing, sitting and moving the spine) with pain drawing and physical examination findings indicating nerve involvement and also with other diagnostic methods of nerve involvement such as electrophysiological methods [72]. Larger and more homogeneous patient samples will add internal and external validity to these studies.

## Conclusion

In this diagnostic study on 61 patients with long-standing nerve root symptoms referred for MRI of the lumbar spine we found that MRI-visible nerve involvement significantly underestimated the prevalence of, and showed weak agreement with, findings of nerve involvement detected in a structured physical examination and a simplified pain drawing. The notion that MRI is the diagnostic method of choice in detecting nerve involvement originating in the spine must be questioned. Factors other than nerve involvement visible on MRI may be responsible for findings of nerve involvement in the physical examination and the pain drawing. Using a structured physical examination and a simplified pain drawing in assessing lumbar spine disorders, especially on patients with "MRI-invisible" symptoms, may indicate that these symptoms are less "non-specific" or psychosomatic than hitherto believed and that the patients may need treatment aimed at nerve involvement.

## Competing interests

The authors declare that they have no competing interests.

## Authors' contributions

BCB, LES, EB and HB designed this study. BCB made the clinical examinations and assessments. EB and HB made the radiological examinations and assessments. BCB prepared the manuscript. All authors read and approved the manuscript.

## Pre-publication history

The pre-publication history for this paper can be accessed here:

http://www.biomedcentral.com/1471-2474/11/202/prepub

## Supplementary Material

Additional file 1 **The simplified pain drawing**. Click here for file

Additional file 2 **The structured physical examination protocol**. Click here for file

Additional file 3 **MRI protocol lumbar spine**. Click here for file

Additional file 4 **Ad Table 3**. Agreement between MRI-visible nerve involvement and findings of nerve involvement detected in the physical examination and pain drawing in assessing the lumbar spine (n = 61) Click here for file

Additional file 5 **Ad Table 4**. Sensitivity, specificity, and positive and negative predictive values of MRI-visible nerve involvement to findings of nerve involvement detected in the physical examination and pain drawing in assessing the lumbar spine (n = 61) Click here for file
