# Supplementary material for: Assessment of nerve involvement in the lumbar spine: agreement between magnetic resonance imaging, physical examination and pain drawing findings
Source: BMC Musculoskelet Disord. 2010 Sep 10;11:202. doi: 10.1186/1471-2474-11-202 (PMC2944219; doi:10.1186/1471-2474-11-202)

#### The simplified pain drawing

#### Drawing of discomfort

Where have you had discomfort and what kind of discomfort? Shade with a led pen ALL areas where you have experienced discomfort the last 3 months. Shade off the blackness according to the severity of the discomfort. Describe the kind of discomfort next to the figure, for example: ache, murmur, tingle, stinging, numbness, pain, cramp, buzz.


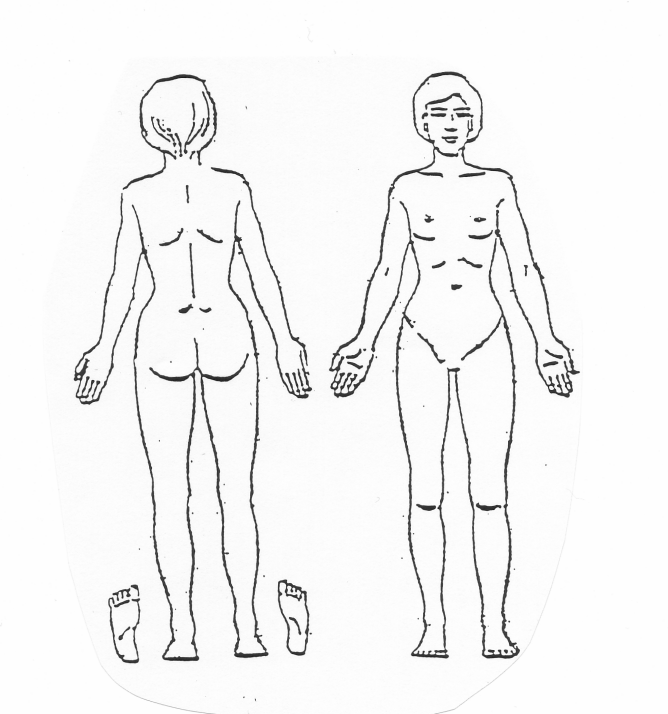

Supplement: Additional file 1 — The simplified pain drawing. [file 1471-2474-11-202-S1.DOC]
