# Supplementary material for: Assessment of nerve involvement in the lumbar spine: agreement between magnetic resonance imaging, physical examination and pain drawing findings
Source: BMC Musculoskelet Disord. 2010 Sep 10;11:202. doi: 10.1186/1471-2474-11-202 (PMC2944219; doi:10.1186/1471-2474-11-202)
Supplement: Additional file 2 — The structured physical examination protocol. [file 1471-2474-11-202-S2.DOC]

**The structured physical examination protocol**
