# Supplementary material for: Assessment of nerve involvement in the lumbar spine: agreement between magnetic resonance imaging, physical examination and pain drawing findings
Source: BMC Musculoskelet Disord. 2010 Sep 10;11:202. doi: 10.1186/1471-2474-11-202 (PMC2944219; doi:10.1186/1471-2474-11-202)
Supplement: Additional file 3 — MRI protocol lumbar spine. [file 1471-2474-11-202-S3.DOC]

**MRI PROTOCOL Patient id:________________________ Patient name:______________________________ Date:_____________ LUMBAR SPINE**

**Examination quality:** **□** Good ______________________________________________

**□** Before reading patient history **□** Suboptimal _________________________________________

**□** After reading patient history **□** Not conclusive _________________________________

| **LEVEL** | **SPINE** | **DISC** | | | **ME-**  **DULLA** | **SPINAL**  **CANAL** | **PROTRUSIO / PROTUBERANCE GRADE** | | | | | | | **RESTRICTION TYPE** | |
| --- | --- | --- | --- | --- | --- | --- | --- | --- | --- | --- | --- | --- | --- | --- | --- |
|  |  | **Water**  **content** | **Height** | **Rim**  **sign** | signal | **Spinal-**  **stenosis** | dx  **extra-**  **foraminal** | dx  **foraminal** | dx  **lat recess** | **MEDIAL**  **paramedial** | sin  **lat recess** | sin  **foraminal** | sin  **extra-**  **foraminal** | **Disk**  Ligaments | **Bone** |
| **Th 9-10** |  |  |  |  |  |  |  |  |  |  |  |  |  |  |  |
| **Th 10-11** |  |  |  |  |  |  |  |  |  |  |  |  |  |  |  |
| **Th 11-12** |  |  |  |  |  |  |  |  |  |  |  |  |  |  |  |
| **Th12-L1** |  |  |  |  |  |  |  |  |  |  |  |  |  |  |  |
| **L1-2** |  |  |  |  |  |  |  |  |  |  |  |  |  |  |  |
| **L2-3** |  |  |  |  |  |  |  |  |  |  |  |  |  |  |  |
| **L3-4** |  |  |  |  |  |  |  |  |  |  |  |  |  |  |  |
| **L4-5** |  |  |  |  |  |  |  |  |  |  |  |  |  |  |  |
| **L5-S1** |  |  |  |  |  |  |  |  |  |  |  |  |  |  |  |
| **(L5-6)** |  |  |  |  |  |  |  |  |  |  |  |  |  |  |  |
| **Grading:** |  | **-** normal  decreased:  **1** slight  **2** significant | **-** normal  decreased:  **1** slight  **2** signif. | **-** absent  **1** present | **-** normal  **1** increa. | **-** absent  **1** slight  **2**signif. | **-** normal  **1** slight  **2** significant | **-** normal  **1** slight  **2** significant | **-** normal  **1** slight  **2** to nerve  **3** deranging  nerve | **-** normal  **1** slight  **2** to dura/  medulla  **3** deranging  dura/med. | **-** normal  **1** slight  **2** to nerve  **3** deranging  nerve | **-** normal  **1** slight  **2** significant | **-** normal  **1** slight  **2** significant | **-** normal  **1** bulging  **2** hernia  **3** sequest | **-** normal  **1** vertebra  **2** interver-  tebral |

COMMENT:

**A** deg. SI change **1** insignif. (inative)

**B** infection (spondylitis) **2** significant (active) ………………………………………………………………….

**C** fracture **-** not applicable

**D** intra osseous disc hernia ………………………………………………………………….

**E** hemangioma

**F** met / destruction ………………………………………………………………….

**G** olisthesis

**H** scoliosis **Radiologist name** ………………………………………………………………….

**X** other (see comment)
