# Supplementary material for: Assessment of nerve involvement in the lumbar spine: agreement between magnetic resonance imaging, physical examination and pain drawing findings
Source: BMC Musculoskelet Disord. 2010 Sep 10;11:202. doi: 10.1186/1471-2474-11-202 (PMC2944219; doi:10.1186/1471-2474-11-202)
Supplement: Additional file 4 — Ad Table 3. Agreement between MRI-visible nerve involvement and findings of nerve involvement detected in the physical examination and pain drawing in assessing the lumbar spine (n = 61) [file 1471-2474-11-202-S4.DOC]

**Ad Table 3. Agreement between MRI-visible nerve involvement and findings of nerve involvement**

**detected in the physical examination and pain drawing in assessing the lumbar spine (n=61)**
